# Evaluation of nursing care associated with infants born to mothers with drugs abuse and its comparison with the standards in selected hospitals in Kerman 2013-2014

**Published:** 2015

**Authors:** Z Mahdavi Khaki, A AbbasZadeh, M Rassoli, F Zayeri

**Affiliations:** * Neonatal Intensive Care Nursing, Faculty of Nursing and Midwifery, Shahid Beheshti University of Medical Sciences, Tehran, Iran; ** Department of Medical Surgical Nursing, Shahid Beheshti University of Medical Sciences, Tehran, Iran; *** Department of Pediatric Nursing, School of Nursing and Midwifery, Shahid Beheshti University of Medical Sciences, Tehran, Iran; **** Department of Biostatistics, Faculty of Paramedical, Shahid Beheshti University of Medical Sciences, Tehran, Iran

**Keywords:** infants born to mothers with drugs abuse, the standard of nursing care, neonatal intensive care

## Abstract

**Background.** Pregnancy of women addicted to drugs is a public health problem in most countries, leading to various problems in the mother, the fetus, and the newborn. Since these babies are at risk of various complications and even death, competent and appropriate care of these children is needed. The present study aimed to assess the quality of nursing care provided to newborns and its comparison with the existing standards in infants and neonatal intensive care units of the selected Hospitals in Kerman.

**Materials and Methods.** In this descriptive conducted study, 400 nursing cares, provided to infants born to mothers with drugs abuse, observed and were compared to standard checklists provided by the latest resources and the world’s scientific papers. The checklist provided was based on the evaluation of infants and included two distinct categories: non-drug therapy and drug treatment. Finally, the data were analyzed.

**Results.** The consistency quality of the nursing cares provided to infants born to mothers with drugs abuse was evaluated with the existing standards in children, 73% receiving non-drug therapy and 81% of the infants receiving drug treatment.

**Conclusion.** Compared to standards in the normal state, nursing care was associated with babies born to mothers with drugs abuse. The reduction in the incidence of morbidity and mortality in this group of infants was expected in the case of familiarity and training of nursing and the use of caring standards, particularly when applying non-drug therapy.

## Introduction

High-risk birth of newborns is one of the major problems discussed in different societies. Despite considering the gestational age or birth weight of more than average, high-risk infants are predisposed to diseases or mortality. Babies born to mothers with drugs abuse include a group of high-risk infants whose complications must be prevented and mortality must be reduced, needing special care and attention [**1**]. Since these infants are at risk of complications and even death, in order to receive medical attention and nursing, the best place to care them is the neonatal intensive care units. In these circumstances, the use of nursing standards leads to the early detection of symptoms, rapid onset of treatment, reduced withdrawal symptoms in newborns and improved neonatal outcomes such as reduced length of stay in the hospital [**2**].

The research carried out the need to the proficiency of the nurses; neonatal intensive care nurses in the care of these infants were especially emphasized. Challenges that nurses in neonatal intensive care units in the care of these babies are faced with include pain management, non-drug measures, drug treatment onset, and care of family members, breastfeeding, birth registration application, and registration of newborns with drugs such as buprenorphine and methadone [**3**]. In this regard, the level of knowledge, patience, commitment, and performance in providing high-quality care to these newborns is effective. Therefore, all the nurses are required to make an efficient use of the care standards and increase education and awareness to reduce complications [**2**]. Nursing and management of these infants require a collaborative teamwork approach and cooperation between the various care groups [**4**].

The pregnancy of women who abuse of substances is one of the major public health issues. Women, who use the material regarding the unwanted pregnancy and prenatal period complications, are the most vulnerable groups [**4**]. At present, the rate of using these materials in women at a childbearing age, which is of about 10.9 percent compared to the past (4.4% in 2010), shows a significant increase [**5**]. 

Since there may be some cases, for fear of being reported to the authorities, they are concealed, but, the real number of these people seems to be far more [**4**].

Substance abuse is also a common practice in Iran and the methods of use are changing to new and high-risk composition of the crystals and injection practices [**6**].

According to the World Health Organization, opium and then heroin are the most common materials in Iran that are used more inhaled [**7**].

Drug use during pregnancy can lead to problems and complications in the mother, fetus, and newborn. Premature birth, miscarriage, placental abruption, and postpartum hemorrhage are the effects of drug use among the pregnant women. Furthermore, these women are at a raised risk of health problems such as malnutrition, anemia, urinary tract infections, sexually transmitted diseases, hepatitis, and AIDS. On the other hand, intrauterine growth restriction, premature birth, and stillbirth are the effects of substance abuse during pregnancy [**8**].

Low Apgar score, meconium aspiration, microcephaly, low birth weight, and Neonatal Abstinence Syndrome (NAS) are the effects of substance abuse during pregnancy that affects the health of the infant after birth [**9**]. Neonatal Abstinence Syndrome (NAS), a condition in which more than half of babies are born to mothers with drugs abuse, is seen during the first few days after birth. It causes central nervous system symptoms (tremor, excitation-taking, seizures, sleep, and muscle stiffness), respiratory tract (apnea, tachycardia, and tachypnea), and gastrointestinal problems (diarrhea, vomiting, dehydration and difficulty in feeding) in newborn [**4**].

Because infant mortality is one of the most important health indicators used in each country, and due to the high mortality rate in this group of infants, using care standards and assessing conformity of cares with standards is necessary to provide high-quality care to babies. Therefore, this study was undergone to determine the compliance rate of nursing considerations in infants born to mothers with drugs abuse with the existing standards, estimating the care distance with these criteria. 

## Materials and Methods

A recent, descriptive study that aimed to compare the current state of presenting cares for infants born to mothers with drugs abuse to the existent standards in the neonatal intensive care units, in selected hospitals in Kerman, during 2013 and 2014.

Regarding that, the aim was to determine and comprise the adaptation ratio of nursing care of infants born to mothers with drugs abuse with the desirable situation. Using the formula of sample size for the rate in a population, the required sample size with a confidence level of 95% and the maximum estimation error of 5%, 385 samples were obtained and by including the loss of a sample size, 400 surveillances were considered as an example for this study.

With a proportional allocation to the amount of births and newborns hospitalization, this sample size was divided among the investigated hospitals. In this regard, the quota for each section was determined as it follows: Afzalipour Kerman hospital: 200 observations were done, that were divided according to the available beds into 134 observations in the neonatal intensive care unit and 66 observations in the neonatal ward. Ayatollah Kashani Hospital in Kerman: 84 observations were performed in the newborn ward. Imam Reza Hospital in Sirjan: 58 observations were conducted in the infants department. Doctor Gharazi Hospital in Sirjan: 58 observations were carried out in the infants department. Except for Afzalipour Kerman Hospital, the other hospitals studied did not have neonatal intensive care units.

In order to prepare a checklist of care standards for the infants born to mothers with drugs abuse, because of the absence of a list of care standards for the offspring of parents with drugs abuse, the level of care of these babies was extracted and collected. For this purpose, the first scientific research papers in the world and developed protocols and guidelines as well as nursing and babies subspecialist reference books that were collected in the years 2013-2005 were analyzed. 

Based on the amount and duration of drug exposure and severity of withdrawal symptoms in mothers, receiving non-drug or drug therapy was required. Various tools were used to determine the type of treatment needed by infants. Finnegan’s Modified Neonatal abstinence scoring system was approved by the Ministry of Health and Medical Education and was used to determine the type of treatment required for these babies. In addition, two separate checklists, which included nursing care associated with infants born to mothers with drugs abuse treatment, needed nondrug or drug therapy, were designed. Check List 1 contained a nursing care with 16 words in the non-drug treatment of babies born to mothers with drugs abuse.

Check List No. 2 provided nursing care with 23 words in the drug treatment of babies born to mothers with drugs abuse. To determine the validity of the checklists, the methods of determining the content validity index and face validity were used to evaluate the reliability of the method and the inter-rater coefficient.

Also, both the time sampling and the sampling of the events method were applied to gather information and nursing care for babies born to mothers with drugs abuse through time sampling was observed and recorded in three shifts: morning, evening and night and then compared with the provided standard checklist.

The information obtained from the checklists resulted from 400 observations of the delivery of nursing care to infants born to mothers with drugs abuse, were extracted. Then qualitative data on the status of the nursing care of the babies were categorized in 3 degrees: conducted right, did wrong and not done, then they were evaluated as quantitative data with code 0, 1 and two respectively in SPSS software.

**Ethical considerations**

The recommendation letter of the Education Deputy of Nursing and Midwifery College in Shahid Beheshti University of Medical Sciences to introduce to Kerman University of Medical Sciences and the Social Security Organization of Kerman was obtained. Then the recommendation letter of such centers to add Afzalipour, Ayatollah Kashani Kermani and Sirjan Doctor Gharazi Hospitals was obtained. At the beginning of the research, the objectives of the study and how it should be done were announced to each authority of the concerned centers. At the request of hospital personnel, the results of the survey were put at their disposal. Scientific articles cited the mentioned source.

Hospital officials and nurses of given sections were assured that the data analyzed, were only for the research and not for any other users, and that, in case of willingness, they would be in the possession of the hospital.

## Results

The frequency distribution of the implementation rate of standards related to nursing care in infants born to mothers with drugs abuse and children in the infant’s wards and neonatal intensive care units for the Hospital of Kerman in 2013-2014, are presented in **Table 1**.

**Table 1:** Frequency distribution of the implementation rate of standards related to nursing care in the Hospital of Kerman in 2013-2014

	non-pharmacological treatment		Drug treatment		p-value
Nursing care	number	percent	number	percent	
Conducted right	117	65.4%	159	71.9%	
Conducted wrong	29	16.2%	41	18.6%	
Not conducted at all	33	18.4%	21	9.5%	
Total	179	100%	221	100%	0.034
Score (percent)	73%	81%			

**Table 1** describes the implementation rate of the appropriate nursing care provided for the non-drug treatment related to infants born to mothers with drugs abuse in the infant wards. Also, according to nursing care checklists, neonatal intensive care units is 73% of which 65.4% of the care was right, 16.2% of the care was wrong, and 18.4% was not conducted. Moreover, according to the **Table 1**, the implementation rate of the optimal nursing care and the neonatal intensive care unit by checklists of nursing care was 81% of which 71.9 was right, 18.6% was not conducted right, and 9.5% was not conducted. According to this table, the highest rate of compliance with care standards was related to checking the list of medical treatment - 81%. Test significance was less than 05/ 0 (034/ 0p =). Hence, the rate of nursing care has a significant difference in the two kinds of treatment.

**Table 2:** Data on infants born to mothers with drugs abuse in infants wards and the neonatal intensive care units in the Hospital of Kerman during 2013 and 2014

	Non-drug treatment		Drug treatment	
	Min	Max	Min	Max
Gestational age in terms of week	36	40	35	40
Weight in grams	2000	3200	1500	2500
Infant age in terms of hours	2	120	5	96
Infant Apgar score	6	9	6	9

Most infants who received a non-drug therapy (53.1%), hospitalized in the infants ward and the neonatal intensive care unit were male and infants receiving medical treatment, and most of the babies (50.7%) were females. Most of the children who received non-drug therapy (65.4%) and most of the babies receiving drug treatment (56.1%), were born through natural childbirth; most of the babies receiving medical care were born by Caesarean section.

**Table 3:** Data regarding the nurses providing neonatal care to infants born to mothers with drugs abuse and infants in the neonatal intensive care units in the Hospital of Kerman during 2013 and 2014

	Non-pharmacological treatment		Drug treatment	
	Min	Max	Min	Max
Nurse age	24	43	25	43
Nurse experience	1	20	3	21
Experience in the neonatal unit and neonatal intensive care	1	15	3	10

Also the level of education in 96.6% of the nurses also providing non-drug treatment and 95.5% of the nurses offering medical care was BA. The study found that 54.2% of the nurses offering non-drug treatment and 66.1% of the nurses delivering care passed the neonatal intensive care training. Also, 92.2% of the nurses providing non-drug treatment and 92.8% of the nurses delivering medical care, employed in the infant’s wards and neonatal intensive care units, were working in circulating shifts.

Besides, 76.5% of the nurses offering non-drug treatment and 71% of the nurses providing medical care, employed in the neonatal wards and neonatal intensive care units, were united. Also, 51.4% of the nurses offering non-drug treatment and 51.6% of the nurses providing medical care, employed in the neonatal wards and neonatal intensive care units, had more than two children.

According to the items viewed in the checklist, the most encountered case of nursing care in non-drug treatment that was done correctly was related to item 1 in the list (prescription by a doctor was precisely checked - 98.9%). Then, the least case that was properly done was related to item 16 in the checklist (baby assessment by using assessment tables, at every 4 hours for at least 72 hours - 0%). Then, item 12 in the list (to calm baby, the swaddle was used - 75.4%) and item 10 (using Kangaroo care was used as a palliative treatment - 50.8%) were done. Also, the most often met case in the checklist, which did not arrange correctly, was related to item 4 (the scoring tables were used at every 4 hours to obtain a score below 8 - 38%). The most often met cases of nursing care in drug treatment that were done correctly were related to item 3 in the checklist (the nurse washed his/ her hands before touching the baby - 99.5%). Also, the least case that was properly done was related to item 16 (baby assessment at every 2 hours and it continued to 24 hours after receiving a score below 8 - 2.3%). Also, the most often met case on the checklist that was not done correctly was related to item 18 (cardiac and respiratory monitoring continued for at least four days after drug withdrawal - 45.7%).

## Discussion

Research findings showed that most neonates’ gestational age observed in both types of treatment presented, was 38 weeks. The result of the study of Torshizi M, Sa’adatjoo A and Farabi M [**10**], which was conducted with the aim of highlighting the prevalence of substance use and its side effects in pregnant women, found that 31% of the women taking the drug had an on time or a preterm birth. Moreover, the birth weight of most infants observed in both types of treatment provided was between 2500 and 3000 kg. In the study conducted by Aziz Mohammadi S and Alavi H [**11**] with the purpose of highlighting the link between substance abuse and pregnancy complications, it was found that the birth weight of infants born to mothers with drugs abuse was lower compared to other infants. In the present study, no comparison was made between the weight of the babies born to mothers with drugs abuse and healthy infants. Most of the children observed in this study had less than 24 hours of age in both treatments provided. Logan B, Brown S and Hayes M study [**12**] with the aim of treatment of neonatal withdrawal syndrome and its results, showed that most of infants born to mothers with drugs abuse in the early hours after birth, needed treatment and protective measures. Most infants, who received non-drug treatment, were males and most infants receiving medical care were females. Most children observed in the non-drug treatment were born, and most infants receiving care were born by Caesarean section. In Torshizi M, Sa’adatjoo A and Farabi M research [**10**], with the purpose of the prevalence of substance use among pregnant women, it was found that 33 percent of the mothers taking the drugs, had a Caesarean section and 66 percent had a natural vaginal delivery. Most neonatal Apgar scores observed in both treatments were 8. Sharifian J et al.’s study [**6**] with the aim of the end of maternal, fetal and neonatal period of pregnant women with substance abuse showed that 83% of the infants born to mothers with drugs abuse had Apgar scores between 7 and 9.

In this study, the average age of nurses providing care in both treatments was between 23 to 33 years. In Goudarzi Z, Khosravi Kh, Bohrani N, Sakooei Kh and Valipour Gavakani V study [**13**] that was conducted in the children sections in Hospitals affiliated to Tehran University of Medical Sciences, the majority of nurses in neonatal intensive care units (NICU) (47.6%) were in the age group of 28 to 31 years. Nurses’ work experience in both types of treatment was between 10 to 15 years. Also, in this study, it was found that the majority of nurses working in the infants-sections and neonatal intensive care units, in both types of treatment had less than five years of experience in the NICU, and most nurses working in the infants-sections and neonatal intensive care units passed the required training courses.

A study conducted by Pritham UA, Paul JA and Hayes MJ [**14**] aimed to assess the substance abuse during pregnancy and the period of withdrawal syndrome in neonates. It was found that there was a relationship between the nurses’ work experience as well as passing the training courses and knowledge and how to provide care, and, with an increase in work experience and passing the courses training, education and practice of nurses and observing the standards of care after birth, will be improved. Most nurses providing care in both types of treatment had a bachelor’s degree. Shiftwork of most nurses providing care in both treatments has been rotating. Most nurses providing care to infants observed in this study were married and had more than two children. Jaloo Z [**15**] conducted a study with the aim of auditing the nursing care of infants with the respiratory distress syndrome hospitalized in neonatal intensive care units in hospitals affiliated to Tehran and Shahid Beheshti Universities of Medical Sciences and Health Services. Their results showed that the level of education of the majority of nurses observed (95.4%), was bachelor.

## Conclusion

Based on the results of this study and examining the items in the checklist, it was determined that in the treatment of infants born to mothers with drugs abuse, the majority of nurses had problems in using standard tools to assess the child. The lack of recognition of the appropriate tools and their proper use, as well as the determination to use non-drug or drug treatment for infants were among the most common problems of the nurses working in infants-sections and neonatal intensive care units. According to the study of Unger A, Metz V and Fischer G [**4**], there were some useful tools to identify infants at risk for drug use. The Modified Finnegan Scoring Table that was recommended by the American Academy of Pediatrics and approved by the Ministry of Health and Medical Education of Iran was an example of the useful tools.

According to the research findings, in the non-drug treatment for infants born to mothers with drugs abuse, the minimal nursing care used for these infants and the assessment of infants by using the scoring table was for 72 hours. According to the protocol approved by the American Academy of Pediatrics, typically every child exposed to substance abuse, should be evaluated at every 2 to 4 hours by using the scoring tables.

This assessment should be continued for 48 to 72 hours after the treatment completion. Also according to the present study, it was found that around 75% of the nurses did not use swaddling to calm infants. In a survey conducted by Kelly et al. in 2011, with the aim of managing and controlling the infant at risk for drug abuse, it was determined that one of the most effective non-drug treatment in these children was the use of swaddle. In non-drug treatment, about 51% of the nurses did not use kangaroo care as a type of palliative treatment. In Dow et al. study it was stressed that Kangaroo care was one of the most effective non-drug therapies in these newborns.

The results of present study revealed that, in providing medical treatment to infants exposed to drugs, about 50 percent of the use of morphine for neonates by using the scoring table was in trouble. According to the study of Dow et al. (2012), the use of the modified Finnegan scoring table and the use of morphine were started in an infant by getting three consecutive equal points, or more than 8 or two consecutive equal points or more than 12. In the medical treatment of babies born to mothers with drugs abuse, about 73% were performed in the neonatal intensive care unit under cardiorespiratory monitoring. In a study by Kelly L, Minty B, Madden SH, Dooley J, Antone I [**16**], it was emphasized that providing medical treatment in infants exposed to the substance abuse should be done in the neonatal intensive care unit under cardiorespiratory monitoring. Also, only about 50% of the infants’ breast-fed continued during the medical treatment. According to the Protocol of American Academy of Pediatrics, breastfeeding could continue during the medical treatment, except for the cases of multi-drug-using by the mother or a mother addicted to crack and heroin. About 45 percent of the infants were not under cardiorespiratory monitoring after the discontinuation of the medical treatment for four days. According to the Protocol of American Academy of Pediatrics, every infant receiving the care should be under cardiorespiratory monitoring for four days after discontinuation of medical care.

According to the results of the research in planning for the infant’s discharge with a clear and comprehensive plan after clinical consulting, about 50% were ineffectively. According to the study of Logan et al. (2013), before discharging the infant, the family’s ability to care for the infant should be evaluated. Also, the use of the fully discharged program and with the presence of experts in several fields related to that group of infants and after a full training of parents to recognize symptoms of withdrawal syndrome could be significantly effective in reducing the babies’ mortality.

The results of this study suggested that the nursing care associated with infants born to mothers with drugs abuse was moderate as far as standards were concerned, which was compatible with the previous studies. The nurses’ lack of knowledge of effective tools and lack of using the tools in the evaluation of the newborns represent the leading causes of low standards of nursing care. Lack of nursing skills on a variety of effective non-drug treatment in treating these infants, as well as the necessity of the start time of medical care in these children represent the practical factors which reduce the effective nursing care in the newborns. Also, the shortage of required facilities and equipment reduces the quality of nursing care services and makes nurses disappointed regarding the implementation of their duties. The lack of education and the lack of in-service training courses for those who provide care and inadequate knowledge, information and skills to provide accurate measures of nursing as well as nurses’ lack of awareness about the importance of a proper care and the consequences of improper care, are of paramount importance. Also, the lack of attention to the standards of care of care providers and the absence of a detailed assessment regarding how the authorities should take care in this regard could be considered among other reasons.

**Limitations of the study**

One of the main limitations of the study was the impossibility of using the laboratory studies to identify infants at risk of substance abuse. All infants under study were identified through the mother declaration of drug addiction or by the emergence of withdrawal syndrome symptoms. The presence of researchers in the field of the study, could lead to changes in performance of the health care providers, thus, whenever the researcher had such a perception of people, he did not register observation, and also he increased the number of presence in the section.

**Acknowledgement**

This article is the end result of one of the authors’ Master in science thesis. The authors would like to thank dear professor Dr. Abbaszadeh for the guidance and efforts and the other professors as well as administrators and nurses in hospitals under study for their help in realizing the study.
